# On the Trial of Mr. Donnell

**Published:** 1817-08

**Authors:** 


*For the London Medical and Physical Journal.*

### *On the Trial of Mr. Donnell;* by Aminicus.

I BEG leave to transmit to you a few remarks on your Report of the Trial of Mr. Donnell, which has also appeared at length in the London newspapers. It is not only very surprising, but much to be regretted, that, in a case wherein the life of an individual was at issue, as well as the interests of humanity deeply involved, every possible means of elucidation which the present advanced state of chemistry could furnish was not had recourse to; for it is well known to chemists that even a *single grain* of the white oxyd of arsenic will (when properly treated) exhibit such metallic appearance as to leave no doubt of its presence. Humid analysis is liable to deceptive appearances from adventitious causes, and therefore ought never to be solely relied on when life is at stake. How the presence of arsenic could be so clearly evinced in a fluid state as to leave "not the slightest doubt of its being the cause of Mrs. Dunning's death," when it could not be reduced to a metallic state, (simple as the process is known to be,) I am at a loss to conjecture.

Reflecting on this trial, I have been led to contrast the conduct of Dr. Edwards with that of the late John Hunter, in the memorable case of Captain Donnellan, who was executed for poisoning Sir Theodosius Boughton by laurel-water. The judicious remarks and extreme care manifested by him while under examination in court, were such as bespoke the character so justly given of that great man. I think his remarks on experiments are worth recording in your valuable Journal.

He says, "I apprehend a great deal depends upon the *mode* of experiment. There is no man fit to make an experiment (under such circumstances) but those *who have made many*, and with a considerable attention to *all the circumstances* that relate to experiment. It is a common experiment, which I believe seldom fails, and is in the mouth of every-body, that a little brandy will kill a cat: I have made the experiment, and have killed several cats; but it is a *false*

*false experiment*, for, in all those cases where it kills the cat, it has got into the *lungs*, and not the *stomach*—if you convey it into the stomach so as not to affect the lungs, the cat will not die," &c. &c.

I have been induced to turn to the evidence given in court of the effects of laurel-water on the stomach in the case of Sir T. Boughton, from the instance of sudden death reported in a monthly periodical work (probably copied from some newspaper) of Mr. Middleton's housekeeper, occasioned by her drinking a quantity of that deleterious article by mistake. This statement, I find, by communication with Mr. M. is very incorrect, and ought, therefore, to be rectified. The case was briefly this:—Mr. M.'s housekeeper was ordered to prepare a quantity of boiled custard, to be ready for company expected on the succeeding day. Four or five laurel-leaves had been employed, as usual, to give it a flavour; and, a small portion of the custard remaining in the pan, after the requisite quantity had been poured out, the housekeeper was induced to taste it, inviting also another female servant to partake with her, which she did. The housekeeper died before medical assistance could well be administered; and the other servant was only saved by the best possible attention being paid to her, she having, it was supposed, taken less of the custard. It is very remarkable that this servant exhibited all the symptoms of drowsiness, inaction of the stomach, &c. which usually succeed the taking a fatal portion of Tincture Opii, for the judicious and well-directed efforts of those around her were scarcely sufficient to prevent her sinking into sleep.

This appears a very remarkable circumstance, inasmuch as it was not manifest, either from the numerous experiments of the late John Hunter, instituted on different animals previous to the trial of Captain Donnellan, or from the effects of laurel-water on the stomach of Sir T. Boughton, that any thing like drowsiness or inaction of the stomach was apparent. On the contrary, he complained to Lady Boughton repeatedly that his medicine occasioned so much sickness that he was afraid of being unable to retain it on his stomach, &c. I am quite at a loss to account for this discordancy of symptoms; but, perhaps, some of your numerous medical contributors, more conversant with the subject, may think it worth while to oblige your readers with their sentiments.

*Doncaster; May* 13, 1817.

---

This gentleman has favoured us with his name, but delicacy induces him to decline its publication.—Edit.

